# On the Therapeutical Employment of the Oxide of Zinc

**Published:** 1861-10

**Authors:** S. Waterman


### ON THE THERAPEUTICAL EMPLOYMENT OF OXIDE OF ZINC.

By S. WATERMAN, M. D.

*From the American Medical Monthly.*

Oxide of zinc is known to be a powerful therapeutical agent, yet its peculiar mode of action upon the human organism, and the various diseases in which it finds a rational application, are by no means yet fully understood. In the United States Dispensatory, the account given of this article, is meagre and unsatisfactory. Some of the European Pharmacological works give a more minute description, but also without, by any means, exhausting the subject. I shall probably not state much that is new to *all* the readers of the *Monthly*, but from the observations which I have had the opportunity of making, I feel called upon to direct the attention of the profession to this medicine.

Oxide of zinc exerts a powerful sedative or soothing influence over the brain and the ganglionic nerves. Whether this influence is produced by direct action of the zinc upon the brain ; whether it acts by a sort of revulsive influence, by transferring cerebral irritation to the plexus of the stomach, it is not possible as yet to tell. Its operations seem to be exceedingly mild, and free from after effects, such as are observed after the use of opium, and the kindred narcotics. In all cases where these narcotics are contra-indicated in diseases of the brain, zinc may be given, and in many of these cases its effects are positive and lasting ; so that I have often considered it worthy of the title of "the opium of the brain." Its ability to soothe vascular excitement, dependent upon cerebral and nervous irritation, cannot be well doubted ; whilst it possesses equal power to allay and calm the irritation of the brain and ganglionic nerves, unaccompanied by inflammatory action of these tissues.

It has proved itself useful to a limited extent, also, in inflammation of the brain and its membranes, but in a manner less marked and positive; and I think it, therefore, les sworthy of confidence in these diseases.

From its action as described above, it is rational to expect beneficial results from its exhibition in the following diseases; and these expectations are borne out by actual trial and observation:

1. In *delirium tremens.* Indeed when all other remedies seemed to fail—when opium and its kindred narcotics had been unable to make any impression, and a fatal issue seemed unavoidable, oxide of zinc has in several instances rendered me such signal service, as fully to justify the confidence I feel in its therapeutical power. It need not be given in large doses even here; two or three grains every two hours are sufficient. It may be combined, if necessary, with opium or other remedial agents; and its use should be continued for some time even after the delirium has been overcome.

2. In *eclampsia infantilis*, after the fits have been broken by the free use of chloroform, zinc will be found a very serviceable agent to control the cerebral and nervous agitation, which almost always still harasses the little patient for a short time. I have often combined oxide of zinc in grain doses, with calomel, digitalis, and pulv. scillæ. Its effects have been thus most gratifying, and the impartial practitioner can certainly readily verify the truth of the observation. As in the former disease, its use ought to be continued after the convulsions themselves have disappeared.

3. In the *eclampsia* happening during pregnancy or labor, or menstrual irregularities, oxide of zinc acts beneficially upon the sensorium, with or without the administration, at the same time, of opium and calomel; especially in those cases where the convulsions originate from a high state of nervous sensibility, as is the case in subjects to hysteria, in its various forms. After the employment of chloroform, zinc will be found to exert its sedative influence in a most positive manner. I have given two or three grains per dose, every two hours, and would not advise a larger dose. In convulsions produced by plethoric habits, its effects are less reliable; whilst in convulsions arising from uræmic toxication, its effects have not been sufficiently tried to warrant any positive statement in its favor.

4. It will be found a most reliable agent in the various forms of *hysteric convulsions*, depending, as they do in most cases, upon inordinate action of the nerves and their centres. It is in these cases that its influence over the plexus of the stomach is most strikingly verified.

5. In the *exanthematic diseases*, when the eruption is accompanied by cerebral irritation, and even by convulsions, oxide of zinc has long been known to exert a soothing effect upon the nervous centres, and has been used by the most experienced European practitioners.

6. I have treated a few cases of *epilepsy* and a few cases of *chorea* with zinc. I have not had sufficient opportunity to watch its influence, but believe it to be, as stated by various practitioners, from time to time, for more than half a century, a very efficacious remedy in some cases; more especially, it is true of chorea than of epilepsy.

7. European writers recommend it as an excellent remedy in asthmatic difficulties and whooping-cough; but I have no experience to offer on this head, nor on the three following.

8. In England and in this country it has been very highly extolled against the night-sweats of phthisis, and as useful in all cases of profuse perspiration.

9. It has also been well recommended against worms, and still more against chronic dysentery.

10. To complete the list, I must yet mention that it is reported to have cured some of the most obstinate cases of intermittent fever.

The external use of oxide of zinc—unsurpassable in its satisfactory results, as it sometimes is—seems to be well enough understood by the profession generally.

In the estimate I have placed on the oxide of zinc, I have relied strictly on the results of my own practice. I have the notes of a number of cases in point, which I withhold from publication, only not to increase the length of this article; and I urge on all physicians to contribute their quota to our knowledge on the subject, to the end that its physiological effects and therapeutical uses, can at least, be definitely determined.

---

A Correspondent of the N. Y. *World* says, of the wounded in the Washington hospitals, after the Bull Run affair, that in consequence of the comparative *state of nature*, in which they lived for months previous to the action, the cases which would otherwise have been obstinate or incurable, are now in a rapid state of convalescence; in fact, to use the attending physician's words, "their wounds heal like those of wild beasts."

